# CREDO: a friendly Customizable, REproducible, DOcker file generator for bioinformatics applications

**DOI:** 10.1186/s12859-024-05695-9

**Published:** 2024-03-12

**Authors:** Simone Alessandri, Maria L. Ratto, Sergio Rabellino, Gabriele Piacenti, Sandro Gepiro Contaldo, Simone Pernice, Marco Beccuti, Raffaele A. Calogero, Luca Alessandri

**Affiliations:** 1 Polytechnic of Turin, Turin, Italy; 2 Department of Molecular Biotechnology and Health Sciences, University of Torino, Turin, Italy; 3 Department of Computer Science, University of Torino, Turin, Italy; 4 Department of Pathology, Boston Children’s Hospital, Harvard Medical School, Boston, MA USA

**Keywords:** Reproducibility, Bioinformatics, Docker, Open science, Software sharing

## Abstract

**Background:**

The analysis of large and complex biological datasets in bioinformatics poses a significant challenge to achieving reproducible research outcomes due to inconsistencies and the lack of standardization in the analysis process. These issues can lead to discrepancies in results, undermining the credibility and impact of bioinformatics research and creating mistrust in the scientific process. To address these challenges, open science practices such as sharing data, code, and methods have been encouraged.

**Results:**

CREDO, a Customizable, REproducible, DOcker file generator for bioinformatics applications, has been developed as a tool to moderate reproducibility issues by building and distributing docker containers with embedded bioinformatics tools. CREDO simplifies the process of generating Docker images, facilitating reproducibility and efficient research in bioinformatics. The crucial step in generating a Docker image is creating the Dockerfile, which requires incorporating heterogeneous packages and environments such as Bioconductor and Conda. CREDO stores all required package information and dependencies in a Github-compatible format to enhance Docker image reproducibility, allowing easy image creation from scratch. The user-friendly GUI and CREDO's ability to generate modular Docker images make it an ideal tool for life scientists to efficiently create Docker images. Overall, CREDO is a valuable tool for addressing reproducibility issues in bioinformatics research and promoting open science practices.

## Background

Reproducibility is a critical problem in the Bioinformatics field [1]. Bioinformatics research often involves analyzing large and complex biological datasets using several computational tools, algorithms, and models [2]. The results of these analyses are used to draw important conclusions about the underlying biology. However, the issue of reproducibility arises when other researchers try to replicate an analysis. One major factor is the sheer complexity and variability of biological data [3]. In addition, the choice of methods and algorithms can also have a significant impact on the results, leading to different outcomes for the same data [4]. Another factor is the lack of standardization and documentation in the field [5]. In bioinformatics, many specialized tools and pipelines are developed by individual researchers or small teams, however, these tools may not be well-documented or easily accessible to others, which can make it difficult for other researchers to understand, reproduce and verify the analysis, as well as identify potential errors or biases. Last but not least factor affecting reproducibility in bioinformatics research is the complexity of installing and managing libraries and packages. This process can be challenging due to the use of multiple tools and packages, each with its own set of dependencies. Sometimes, different packages may even share the same dependence but require different versions, making it difficult to ensure compatibility. Consequently, it is crucial to carefully document and share the specific versions of libraries and dependencies used in the analysis to improve the reproducibility of the study. To address these challenges, the scientific community has been focusing on improving reproducibility in bioinformatics research [1, 6, 7].

One effective strategy for improving reproducibility is to adopt open science practices, such as sharing data, code, and methods with others, as well as publishing detailed and transparent descriptions of the analysis. Among various tools and platforms available for code sharing and collaboration, GitHub is widely used by researchers in the field of bioinformatics.

Github is a platform for hosting and sharing code, which makes it easier for other researchers to access and use the same tools and workflows. GitHub provides several features that are particularly useful for bioinformatics, such as version control, which ensures that the code remains consistent and can be tracked over time. In this way, GitHub facilitates collaboration and improves the reproducibility of bioinformatics research.

Many bioinformatics tools and workflows are already shared on GitHub, including the Bioconductor project [8], a repository of R packages for bioinformatics [9], and the Snakemake workflow management system, which provides a robust platform for sharing workflows and improving reproducibility [10]. By utilizing GitHub to host and share these tools, researchers can collaborate more easily and ensure that their work is reproducible and accessible to others in the field. Therefore, GitHub has become an essential platform for advancing open science and reproducibility in bioinformatics research. It's important to note that while GitHub is a popular choice for hosting and sharing files, users have the freedom to upload their files to any file-sharing service of their choice.

In addition to code sharing and collaboration, another crucial aspect of improving reproducibility in bioinformatics research is the use of containerization technologies like Docker. Docker provides a way to package software and its dependencies into standardized, portable containers. These containers encapsulate the entire computational environment required to run a specific analysis, including the operating system, libraries, and tools.

The use of this technology offers several benefits for reproducibility:

1. It ensures that the analysis can be executed in the same environment regardless of the underlying system or infrastructure. This eliminates the common issue of software compatibility and dependency conflicts that often hinder the reproducibility of bioinformatics analyses. By sharing the Docker container alongside the code and data, researchers can provide a consistent and self-contained environment, enabling others to reproduce the analysis with ease.
2. Docker enables the preservation of the software stack used in the analysis, including specific versions of libraries and dependencies. This information is crucial for accurately reproducing analytical results, as different versions of software can produce different outcomes. By specifying the exact versions of software in the Docker image, researchers can ensure that others can replicate the analysis precisely, even if the original software versions become outdated or unavailable.

Moreover, Docker containers facilitate the distribution and deployment of complex bioinformatics workflows and researchers with it, can create portable and executable workflow descriptions, known as Dockerfiles, which define the steps required to build a container with the necessary software and dependencies. These Dockerfiles can be shared on platforms like GitHub, allowing others to easily reproduce the entire analysis workflow and obtain consistent results.

The adoption of Docker in bioinformatics has gained momentum, with numerous bioinformatics tools and pipelines now available as Docker images on platforms like Docker Hub and BioContainers. By leveraging Docker alongside platforms like GitHub, researchers can enhance the reproducibility of their bioinformatics analyses by providing a complete and reproducible computational environment for others to utilize.

Reproducibility is a crucial aspect of scientific research, serving two main purposes: ensuring the accuracy and truthfulness of the findings and facilitating the reuse and adaptation of existing analyses.

The ability to reproduce analyses allows for the verification and validation of research results. Reproducibility enables other researchers to independently assess the methods, data, and conclusions of a study, thereby enhancing the transparency and reliability of scientific findings. By providing detailed documentation and clear instructions, including the specific software versions, data preprocessing steps, and parameter settings, researchers can enable others to replicate their experiments and validate the reported outcomes. This process of verification is essential for building a robust scientific knowledge base and establishing a solid foundation for further research and discovery.

Moreover, reproducibility allows the reuse and adaptation of existing analyses, which can save time, effort, and resources for researchers. Often, a new study builds upon previous work or seeks to apply similar methods to a different dataset or research question. In such cases, having access to reproducible analyses provides a starting point and reference framework that can be readily adapted and customized. Researchers can leverage the knowledge and expertise embedded within reproducible analyses to accelerate their investigations, explore new hypotheses, and make further scientific advancements.

However, achieving reproducibility in bioinformatics analyses is not without challenges. The complexity of the bioinformatics landscape, with diverse architectures, operating systems, and software environments, poses significant hurdles since different operating systems or hardware configurations may result in variations in the analysis outcomes. Moreover, the dependency on specific software packages and their versions further complicates reproducibility, as different versions may introduce changes or compatibility issues that can affect the results.

Docker is an open-source containerization platform that allows developers to package applications and their dependencies into portable, lightweight containers. These containers can be easily deployed across different computing environments, including local machines, cloud servers, and clusters. Docker provides a standardized way to create, share, and run software applications in isolated environments, without worrying about conflicts or dependencies on the underlying system. Docker containers are based on a layered file system, which makes them efficient, flexible, and easy to manage. Docker has become a popular tool in many fields of computer science, including software development, data science, and bioinformatics [11].

Ensuring the reproducibility of Docker images remains a significant challenge in the software development community. While Dockerfiles provide a series of instructions for constructing Docker containers, they do not always guarantee the container's reproducibility.

Docker plays a vital role in addressing the reproducibility challenge, providing a standardized and isolated environment where researchers can pack their entire pipeline, including the software tools, libraries, and dependencies, into portable containers. These containers encapsulate the entire computational environment, eliminating conflicts with the underlying system and enabling consistent and reproducible analyses across different platforms. By using Docker, researchers can ensure that their analyses are not only reproducible on their machines but also on other systems, including local computers, cloud servers, and high-performance computing clusters.

However, it is important to note that Docker's reproducibility is reliant on the availability of Docker images, which are typically uploaded to platforms like Docker Hub. While Docker Hub is the official repository for sharing and accessing Docker images, it does have limitations, particularly for non-premium users who may face time restrictions on the availability of their images. This can pose challenges in the long-term reproducibility of analyses. Additionally, the Dockerfile's dependencies and package versions may be insufficiently specified, leading to broken links during the container building process or containers that differ from those created months earlier. A precise and buildable Dockerfile is critical to ensuring container reproducibility at any time, enabling personalized and customizable containers. Therefore, best practices for Docker image creation and distribution should prioritize precise and well-defined Dockerfiles. This involves incorporating heterogeneous packages and environments, such as Bioconductor [8] and Conda [9].

Here, we present CREDO, a friendly Customizable, REproducible, DOcker file generator for bioinformatics applications, which represents a tool moderating the reproducibility issue encountered by building and distributing docker containers embedding bioinformatics tools.

## Results

### CREDO features

CREDO focuses on achieving 100% reproducibility by emphasizing offline building of Docker images, reducing reliance on external platforms such as Docker Hub. This process involves two distinct parts within the tool. The first part, known as "docker image assembly", downloads all the necessary components required for the second part. The second part can be executed offline, as it has all the required files for the complete building process. This offline capability ensures that researchers have full control over the reproducibility of their analyses, regardless of the availability or limitations of external resources. By providing a customizable and personalized Dockerfile generation tool, CREDO enables researchers to include explicit versioning and dependency specifications, mitigating the risk of variations and inconsistencies in the resulting environment.

Moreover it facilitates the process of constructing and personalizing a Dockerfile, enabling users to choose between a command line interface or a user-friendly graphical interface. Docker is undoubtedly the most effective choice for building a tool like CREDO to achieve reproducibility, while maintaining ease of use since it is widely adopted for virtualizing environments and is available on all major operating systems under the Apache license, which reinforces our belief that backward compatibility will not be compromised in future updates. Furthermore, the commands utilized in our tool are built upon the Docker engine, and upon reviewing the Docker deprecated features page (https://docs.docker.com/engine/deprecated/), none of these essential commands have ever been modified. In the unlikely event of a backward compatibility issues, we will take the responsibility of developing a porting tool to ensure a smooth transition of Dockerfiles to newer versions.

The output generated by CREDO includes a comprehensive folder that contains both the Dockerfile and all the required files that have been pre-downloaded for building the Docker image. This meticulous approach guarantees that the Docker image can be constructed reliably and consistently, eliminating the potential issues of broken links or missing library versions that may introduce variations in the resulting environment. By encapsulating all necessary components within the folder, CREDO ensures that researchers can effortlessly construct the Docker image at any given time, thereby enhancing reproducibility and minimizing discrepancies in the analysis environment.

In this context, it is worth mentioning BioContainers, which is a community-driven project aiming to provide bioinformatics software in ready-to-use images. BioContainers offers a curated collection of Docker and Singularity images for popular bioinformatics tools, ensuring reproducibility and ease of use for researchers, providing pre-configured environments with the necessary dependencies, making it easier to run bioinformatics software consistently across different systems.

Another relevant platform in the bioinformatic field is usegalaxy.eu. UseGalaxy is an online platform that offers a user-friendly interface and a vast collection of bioinformatics tools and workflows. It leverages Docker images to provide a seamless and reproducible environment for performing bioinformatics analyses. By utilizing Docker-based tools and workflows available on usegalaxy.eu, researchers can benefit from a unified and reproducible computational environment for their analyses.

However, it is essential to consider the limitations of the above mentioned infrastructures when evaluating their suitability for reproducibility and customization. While biocontainers.pro and usegalaxy.eu offer a vast collection of pre-built images, one notable limitation is the restricted ability to modify and customize them according to specific research requirements. Indeed they are typically provided as downloadable images, along with their respective Dockerfiles. However, due to the nature of their construction and deployment, modifying them, to incorporate additional tools or tailor them to specific experimental setups, can be challenging. Moreover, the reliance on online libraries and dependencies within these pre-built images poses additional concerns. Frequently, the versions of libraries and dependencies are not explicitly specified in the Dockerfile, leading to potential inconsistencies and variations during the building process. This lack of version control can result in different docker images being generated, deviating from the ones hosted on biocontainers.pro or usegalaxy.eu. Consequently, reproducing an experiment exactly as intended becomes more challenging, as even slight differences in library versions can have significant implications on the final results. In contrast, CREDO addresses these limitations by providing a framework that allows for the creation of fully customizable and reproducible Docker images. By utilizing Docker's flexible and robust containerization technology, researchers have complete control over the images composition, including the selection of specific tools, libraries, and versions. Our tool emphasizes the inclusion of explicit versioning and dependency specifications within the Dockerfile, ensuring transparency and reproducibility.

Not only does CREDO provide a reproducible infrastructure, but it also offers a convenient method for distributing complete computing analysis workflows. The compatibility of files generated by CREDO extends beyond GitHub and can be used with any preferred file-sharing platform. This flexibility allows researchers to select the most suitable file-sharing service based on their specific needs and preferences. Additionally, the CREDO output is compatible with GitHub's guidelines and can be directly uploaded to the platform, as all files are split into archives smaller than 25 MB. The Dockerfile built by CREDO is programmed to unpack these archives and reuse them, ensuring seamless integration and ease of use for researchers.

## Implementation

**Fig. 1:**
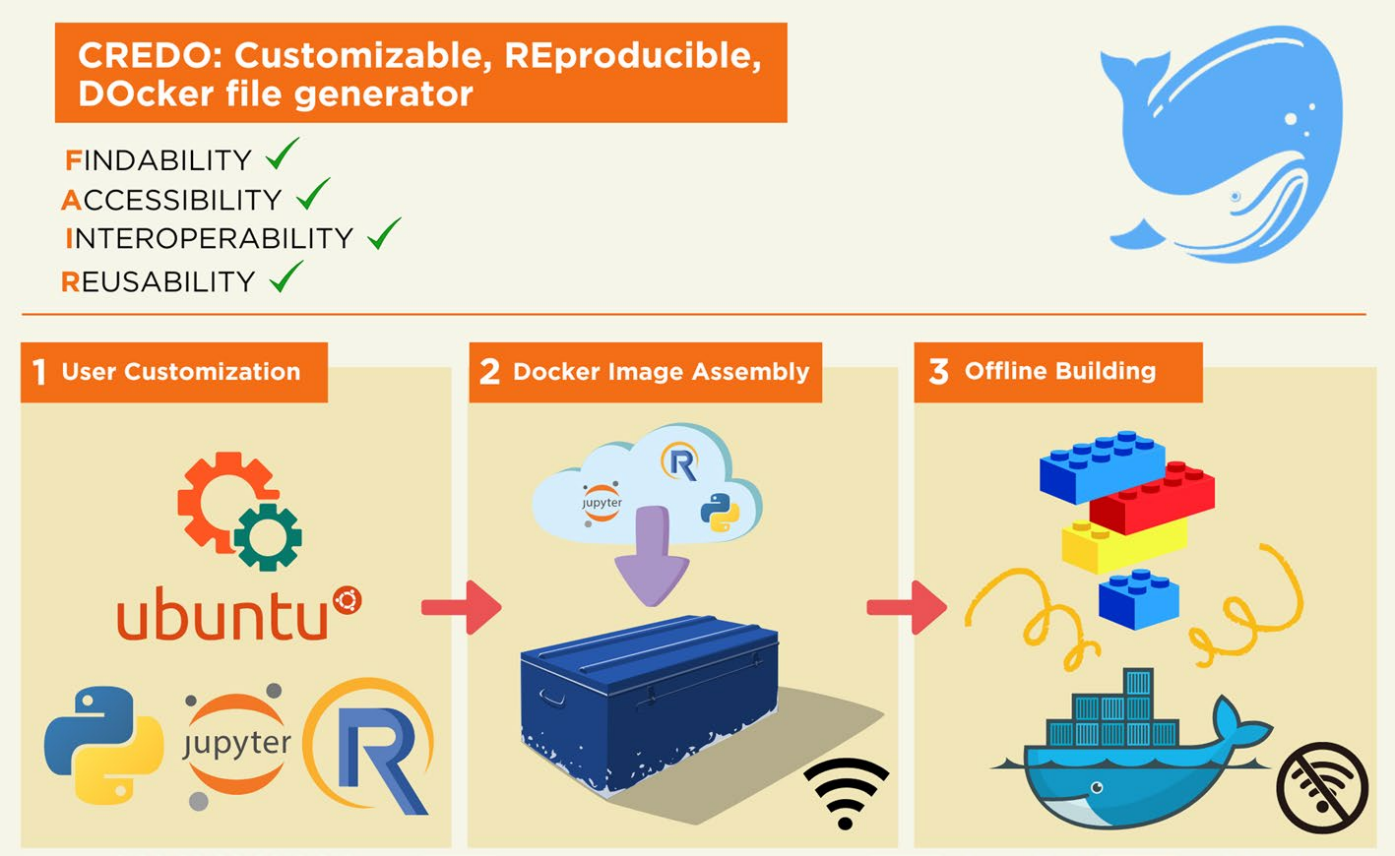
CREDO workflow. CREDO embraces the principles of FAIR, which stand for Findable, Accessible, Interoperable, and Reusable. These principles serve as a framework to promote data and resource sharing in a way that maximizes their usability and impact. (1) User Customization: Represents the first step where users can customize the contents of the Docker image according to their specific requirements. (2) Docker Image Assembly: Represents the second step where a dummy Docker container is downloaded, archiving all the necessary files for offline building, and generating an installation script. (3) Offline Building: Represents the third step where a new Docker image is created using the recorded instructions from the Docker Image Assembly step and the downloaded files. This step ensures reproducibility and independence from internet connectivity, allowing users to build Docker images offline with all the necessary dependencies and configurations

To ensure reproducibility and avoid issues related to broken links or missing package versions, CREDO employs a two-step process, Fig. 1.

Step 1, Fig. 1.2, (docker image assembly): In this initial step, CREDO installs all the required libraries and dependencies in a temporary Docker container, which serves as a “dummy” environment. During this installation process, CREDO captures crucial information about the dependencies, their installation order, and the associated files. This information is then saved and used to generate a script.

Step 2, Fig. 1.3, (docker image offline building): The generated script from Step 1 is integrated into the Dockerfile. This script contains the necessary instructions to install the files offline, based on the acquired information. By incorporating this script into the Dockerfile, CREDO ensures that the Docker image can be built without encountering any issues related to outdated or missing packages. This offline installation feature is particularly significant in scientific applications where reproducibility is crucial.

In summary, CREDO's process involves first downloading and installing all the dependencies in a temporary Docker container to capture the necessary information such as dependencies and to collect all the files in an organized archive. Using these information CREDO generates a script that will be integrated in the dockerfile for the second step. This approach guarantees reproducibility and mitigates issues arising from broken links or missing package versions.

### Repository structure

The CREDO project is divided into two repositories, CREDOengine and CREDOgui, to address the specific requirements of its user communities. CREDOengine is designed for developers and bioinformaticians, demanding deep control and customization capabilities, offering backend code essential for sophisticated Dockerfile generation and management. CREDOgui is designed for users who prefer a more user-friendly entry point, featuring a graphical interface that simplifies Docker image management and creation. This is particularly advantageous for researchers with different levels of computational expertise. Within the Docker build for CREDOgui, users have the option to change the repository path of CREDOengine. This feature allows for the creation of a personalized CREDO instance, enabling users to adapt the tool according to their specific project requirements or preferences, pointing to their forked github page.

### CREDOengine

**Fig. 2:**
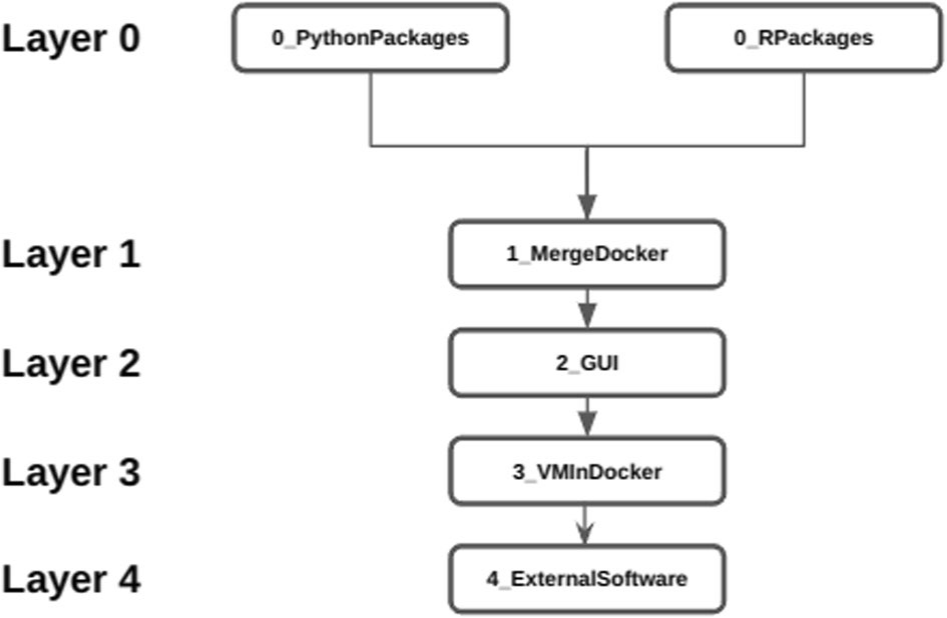
CREDOengine's structured flow across layers. Each layer builds upon the previous one, creating a sequential enhancement of the docker object: Layer 0 provides the basic environment, requiring either Python or R modules. Layer 1 builds on this, adding combined Python and R support, and each subsequent layer extends the capabilities. Layer 2 allows the implementation of a graphical interface. Layer 3 implements the ability to run docker in docker. Layer 4 provides the infrastructure for the installation of additional software beyond Python and R. This sequential flow ensures a coherent build-up of features, allowing users to develop a Dockerfile progressively tailored to their needs

The architecture of CREDOengine employs a layered approach, which provides a flexible and customizable environment, Fig. 2. Layer 0 is designed to establish the basic environment and settings the necessary elements for the other layers. It includes essential packages and setups, such as pre-installed Python or R packages, creating a ready-to-use infrastructure for further customization and tools integration.

Each additional layer adds further capabilities and options for customization. For instance, Layer 1 integrates multiple languages (R and Python in the same docker), while Layer 2 adds user interfaces or additional functionalities. This hierarchical structure allows users to precisely tailor the CREDO enviroment according to their specific needs and technical expertise.

**Fig. 3:**
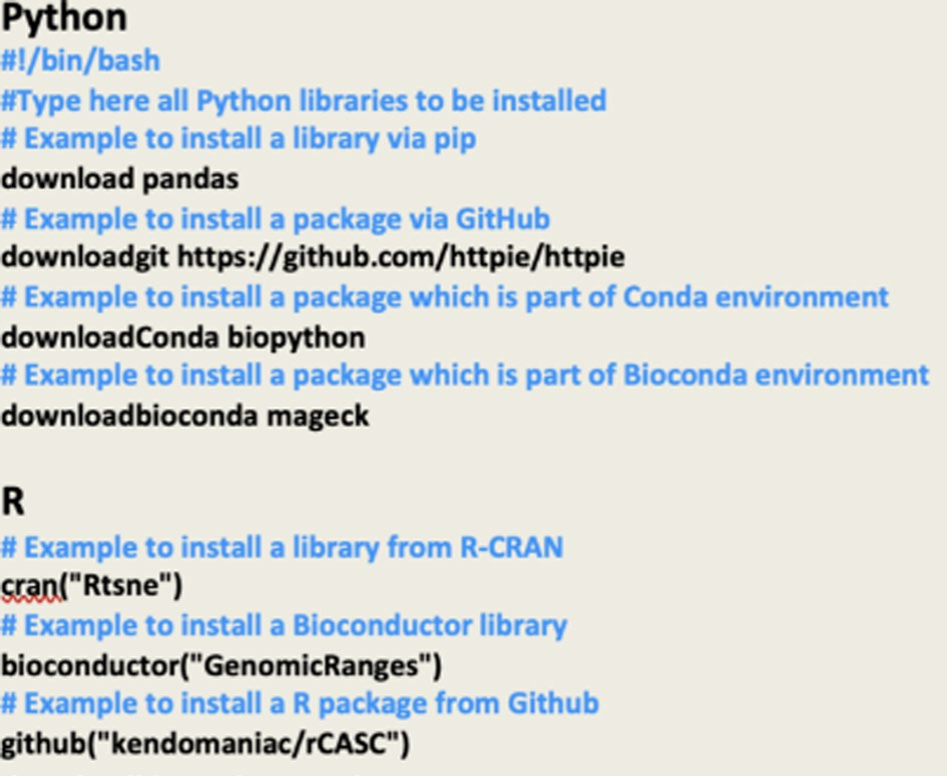
Examples of config files

- Layer 0 generates Docker images that include pre-installed Python packages (0_PythonPackages) or R packages (0_RPackages). This approach provides flexibility in selecting the programming language for developing bioinformatics tools. In Fig. 3, various commands for installing the libraries are shown. To modify the installation process, users can access the configurationFile.sh file for Python or configurationFile.R for R within the CREDO engine. In the user-friendly CREDO GUI, customization can be achieved simply by clicking on the "config" button. These options allow researchers to tailor the Docker image to their specific requirements, ensuring a personalized and reproducible environment for bioinformatics analyses.
- Layer 1 (1_mergeDocker) allows users to merge the Python Dockerfile with the R Dockerfile, previously generated at Layer 0.
- Layer 2 provides a range of programming graphical interfaces (Jupyter lab, Jupyter notebook, Rstudio, and Visual Studio), all of which can be accessed through a web application (http://localhost:8888).
- Layer 3 (3_VMInDocker) configures the virtual environment to execute docker or singularity instances within a docker container. This specific feature is useful in case the running docker container requires executing the software embedded in another docker or in a singularity instance, e.g. the docker used to generate the dockerfiles in CREDO.
- Layer 4 (4_ExternalSoftware) addresses the need for installing additional software beyond Python and R packages in the CREDO framework. Similar to the first layer (layer 0), this one utilizes a configuration file where the names of the software to be installed, e.g. BWA, SAMtools, etc., can be specified as comma-separated values. Currently, this layer only supports the installation of software through the apt package manager.

CREDO offers a more reliable and robust approach for building and distributing Docker images, which is particularly important for bioinformatics applications and other complex workflows. An essential feature of CREDO is its capability to install not only libraries available on standard archives such as CRAN for R and pip for Python, but it can also handle other repositories like GitHub, Bioconductor, Conda, and Bioconda. This ability provides researchers with greater flexibility in installing and utilizing a wide range of libraries and packages relevant to their scientific research. By supporting multiple installation sources, CREDO enhances the usability and efficiency of research workflows, which is crucial in the bioinformatics field, which requires complex workflows and specialized libraries. As previously explained CREDO, saves all information related to the dependency tree and downloads all necessary dependency packages,packages are then divided into files that are sized appropriately for uploading to a GitHub repository, i.e. max 25 MB/each. In a future update, we will let the user customize this value to extend the compatibility beyond Github. Once the dependencies have been detected and downloaded, the temporary Docker container is deleted. A Dockerfile is then generated using the complete list of previously downloaded packages. By creating a folder that contains all necessary files, CREDOengine enables a Docker build to be performed from scratch without requiring any new downloads. This eliminates the risks associated with broken links, changes in package names, and uncontrolled changes in library versions. Python and R are compiled from their source code.

To perform a Docker build, user can run the script.sh/ script.cmd file, which is included in the folder created by CREDOengine. This folder contains all the necessary elements to build the Docker image that was defined.

After the Docker image has been built, the script.sh/script.cmd can be transferred to the designated folder that will be mounted as “shared folder” in the Docker container. This shared folder is located at /sharedFolder within the Docker container, but its location can be easily modified by adjusting the script.sh/ script.cmd file.

The output generated by CREDO is organized into separate folders, each containing a script.sh/script.cmd file responsible for building and running the corresponding Docker image layer. After running the script.sh/script.cmd for the initial two layers (0 and 1), the user is directed to the Docker instance through a bash command line. For layers 2 and 3, the user can access the running container via a web application, at http://localhost:8888, following the execution of the script.sh/script.cmd. This output organization approach enhances the accessibility of the running container and facilitates user interaction, making it an essential feature for scientific applications.

CREDOengine can also be modified, adding or modifying layers, or changing software versions. Each layer is indeed identified by a number followed by an underscore, such that all folders referring to layer 0 start with "0_". In each folder, the script that generates the Docker image must be called "runMe.sh". This is the script that runs the dummy container and downloads and installs all the required packages. In the "0_RPackages" folder of CREDOengine, there is a skeleton file that provides an example of how to add layers for new programming languages or add new versions of R or Python. The configurationFile.txt contains the hostPath, where the results of the CREDOengine will be stored.

### Accessing to Conda/Bioconda environments

Conda Environment are stored into /snowflakes/condaName folder of the docker containers and can be activated with the following code:

source /snowflakes/condapackageName/bin/activate.

Bioconda environment is stored into /snowflakes/biocondaName folder of the docker container and to activate it is enough to run the following code:

source /snowflakes/biocondapackageName/bin/activate.

### Accessing to programming environment GUIs

The programming environment GUIs, which can be added to the Docker image, are: i. Jupyter (Lab or notebook), ii. Rstudio or iii. visual studio. Only one of these programming environment GUIs can be added to the final Docker image.

### CREDOgui

**Fig. 4:**
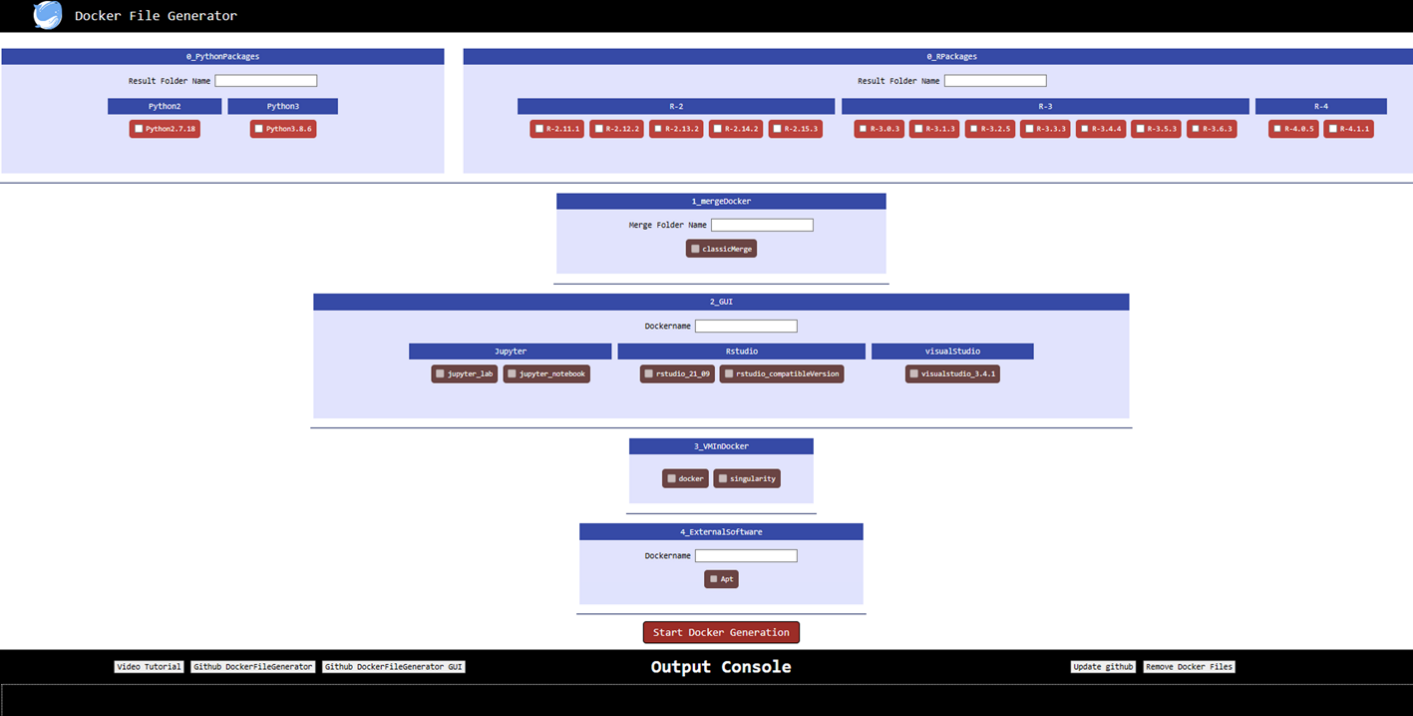
Screenshot of the CREDOgui. In CREDOgui, the dependencies among the different layers are more stringent than in CREDOengine. Specifically, any layer depends on the previous one

CREDOgui streamlines the configuration of the different layers in CREDOengine, as depicted in Fig. 4. It can be accessed through a web application, http://localhost:3000, by running the dockerFileGenerator.sh script for Linux/OSX users or the dockerFileGenerator.cmd script for Windows users, which are both available on the CREDOgui GitHub page. For layer 0, Python and R libraries, to be installed, can be specified using the "Config" button. In layer 1, the user must designate the folder name for the merged Dockerfiles. In layer 2, the user must choose the final name of the Docker image, which is mandatory to be in lowercase and cannot contain any numbers or special characters, and should select a preferred GUI. The name of the output folder for Layer 2 will incorporate the name of the selected GUI. Once the Docker image is built and running, the embedded GUI can be accessed via a web application, http://localhost:8888. In layer 3, it is feasible to install either a Docker or a Singularity instance in the created Docker image and Layer 4, as for layer 0 let the user choose to install other software through apt ubuntu command. In CREDOgui, each layer is reliant on the previous layer, implying that layer 3 cannot be selected unless layers 0, 1, and 2 have been previously configured.

Each layer is self-contained, which means that a user can create a docker image with just layer 0 and layer 1, without selecting layers 2 and/or 3. Once the configuration files are edited and the settings are completed, the generation of the Dockerfile can be initiated by clicking on the "Start Docker Generation" button. The output console provides a summary of the ongoing steps, while a detailed description is available in the log file saved in the folder where the dockerFileGeneration.sh (for Linux and MacOS) or dockerFileGeneration.cmd (for Windows) script is executed.

To enhance the user-friendliness of CREDOgui, we provide a video tutorial that demonstrates how to use the GUI. The link to the tutorial is available in CREDOgui, as shown in Fig. 4 and in the GitHub CREDO readMe. In addition, CREDOgui has two buttons that allow users to access the GitHub pages of the CREDOengine and CREDOgui. There is also a button to download the latest version of CREDOgui (update GitHub) and a button (Remove DockerFiles) that deletes the temporary layers created during the creation of the final Dockerfile.

### Add layers

As pointed out in the previous section, CREDOengine can be modified and those changes are connected to the user interface. These are the steps to follow to add layers to CREDOengine and connect them to the user interface:

1. Create a fork of the repository alessandriLuca/CREDOengine (github.com) and alessandriLuca/CREDOgui (github.com)
2. In the CREDOengine repository, add a new folder for each layer you want to create. Each folder should be named with the corresponding layer number (e.g., 0_pythonPackage, 1_mergeDocker, 2_GUI).
3. Each layer folder can have subfolders to provide options, as seen in layer 2_GUI.
4. In the main layer folder an “inputconfig.txt” file is required. The file needs to have the following structure:
  Temp Docker:H+R
  Result Folder Name:U+empty
  sharedpath:H+/sharedFolder/
  configpath:H+/sharedFolder/configurationFile.txt

Each line of this file represents an input parameter passed to the runMe.sh script. For each parameter, it is necessary to specify the parameter name and its characteristics using the following structure:

- H: the parameter is not shown in CREDOgui
- R: the parameter is assigned randomly from a list of names (see the /nodejs/support/inizialize.js file)
- U: the parameter appears as a box on CREDOgui
- empty: the parameter box will be empty

If the parameter is not random or empty, you can add new values using the “ + ” sign. Note that CREDOgui runs a Docker in Docker, where the shared folder of the physical host and the shared folder of CREDOengine must have the same name but different paths.

(5) Each subfolder (or main folder if there are no subfolders) should contain a "runMe.sh" script, which is responsible for creating the Docker image. The script performs the following steps:
  - Installs the programs in a temporary Docker container, downloading all the necessary files.
  - Saves the files and generates a script that will be integrated into the Dockerfile. This script installs the chosen packages/software from the previously downloaded files.
(6) Modify the Dockerfile in the CREDOgui repository by changing the GitHub clone path to use your forked version of the CREDOengine repository.

These steps allow to add new layers to CREDOengine and connect them to the user interface in CREDOgui.

### FAIR principles

CREDO embraces the principles of FAIR data and promotes reproducibility in bioinformatics research. The FAIR principles advocate for data and methods that are Findable, Accessible, Interoperable, and Reusable. By following these principles, CREDO ensures that the generated Dockerfiles and associated files are easily discoverable and accessible, facilitating their reuse and enabling researchers to build upon existing work. The use of standardized and well-defined Dockerfiles in CREDO enhances interoperability, allowing researchers to seamlessly integrate and combine different bioinformatics tools and workflows. Furthermore, CREDO's approach of incorporating diverse package repositories, such as GitHub, Bioconductor, Conda, Bioconda and apt ubuntu packages, contributes to the availability and accessibility of a wide range of libraries and packages. By adhering to the FAIR principles, CREDO promotes transparency, collaboration, and the advancement of scientific knowledge in the field of bioinformatics.

- Findability: To enhance the findability of our research, we have implemented GitHub as a central platform for hosting our images and software. This consolidation simplifies the research process by providing a unified location for researchers to access, install, and utilize their software. In addition, we plan to introduce a user-friendly structure that allows efficient CREDO utilization and enables DOI associations for Dockerfiles, further enhancing the findability aspect.
- Accessibility: We recognize that the installation of bioinformatics tools or pipelines can be a complex task. In addressing this challenge, CREDO aims to simplify the process by creating Docker images that are accessible even to individuals without extensive bioinformatics expertise. This approach ensures that the code is easily downloadable and usable for both reviewers and researchers interested in utilizing the tool.
- Interoperability: In order to achieve optimal interoperability, CREDO is fully committed in providing Docker images which are compatible with diverse operating systems and platforms. CREDO’s Docker images are carefully crafted to seamlessly function across different environments, enabling researchers to utilize them regardless of their specific setup. To accomplish this, we have implemented various measures, such as incorporating the "–platform linux/amd64" option in the Docker run command. This option ensures that all Docker containers operate with the same architecture, thereby enhancing compatibility across systems. Moreover, CREDO generates a script that automates the building and execution of the Docker image. This script streamlines the process for users, making it transparent and seamless. By encapsulating these compatibility considerations within the script, users can focus on their research tasks without being burdened by technical complexities. Our dedication to interoperability extends beyond technical aspects. We acknowledge the significance of integrating our research tool into existing workflows and collaborations. By ensuring compatibility and interoperability, our future releases aim to facilitate the effortless integration of CREDO into various research environments, enabling researchers to effectively leverage its capabilities.
- Reusability: CREDO focuses on creating Docker images that are rebuildable and reproducible over time. By capturing detailed installation instructions within the Dockerfile, we facilitate modifications and customization of the Docker image. This flexibility allows users to adapt the Docker image to their specific needs and ensures that even if certain libraries become inaccessible online, the Docker image remains self-contained and reproducible. Moreover is it possible to easily customize even the GUI, since it automatically detects any changes in the folder.

## Conclusions

Reproducibility is critical in bioinformatics research to ensure the integrity of findings and facilitate knowledge transfer and collaboration. Docker images, with their standardized and isolated environments, offer an optimal solution for achieving reproducibility. However, challenges such as diverse architectures, different software versions, and reliance on external platforms need to be addressed. Tools like CREDO aim to overcome these challenges by providing customizable and reproducible Docker image generation, promoting transparency, collaboration, and long-term reproducibility in bioinformatics research field. Using CREDO, researchers can easily install the docker image through a graphical interface and only requiring a web browser, providing a seamless experience for working with Docker images and their associated computational environments.

Moreover, CREDO also offers compatibility with GitHub, allowing users to store, version control, and share their Dockerfiles and associated files. This integration enhances collaboration and simplifies the process of sharing reproducible bioinformatics workflows among researchers.

Overall, CREDO empowers bioinformaticians to achieve a high level of reproducibility in their research by generating Dockerfiles that accurately capture the computational environment.

CREDO is indeed a valuable tool in bioinformatics, providing a high level of reproducibility with its Dockerfile generation. Currently, CREDO relies on the availability of the Ubuntu image in the cloud to achieve this level of reproducibility. With the upcoming update even higher reproducibility will be provided by including the base ubuntu image as part of CREDO. In this way, CREDO will provide researchers with greater control and reliability in building Docker images. Combined with its compatibility with GitHub (and similar services) and its user-friendly GUI, CREDO streamlines the process of sharing reproducible bioinformatics workflows, advancing research in the field.

In an upcoming update, we are planning to enhance reproducibility even further. We are actively working on integrating the slim toolkit into CREDO, aiming to optimize the size of the Docker images. This optimization will enhance the efficiency and performance of the generated images, reducing their overall footprint. We are actively exploring the possibility of integrating CREDO with Dataverse, a data repository platform. This integration will provide researchers with the capability to register and publish the Docker images generated by CREDO directly within Dataverse. By enabling this integration, we aim to facilitate the sharing and reproducibility of bioinformatics analyses, fostering collaboration and data accessibility within the scientific community.

As part of the future perspective, we plan to transform CREDO into a hosted service on our University cloud. In the cloud configuration CREDO will provide a multi-user interface, allowing researchers to collaborate seamlessly within the CREDO platform.

Furthermore, we are also exploring the opportunity to integrate CREDO within the European Open Science Cloud (EOSC https://www.eosc-life.eu/) in https://workflowhub.eu/. This integration would enable CREDO users to share and upload the generated Docker images directly to EOSC, facilitating access and resource sharing within a broader research community.

## Availability and requirements

- Project name: CREDO.
- Project home page: https://github.com/alessandriLuca/CREDOengine; https://github.com/alessandriLuca/CREDOgui.
- Operating system(s): Linux, MAC OSX, Windows 10/11.
- Programming language: R, Python, Bash.
- Other requirements: Docker desktop.
- License: GNU GPL.
- Restrictions to use by non-academics: licence needed, please contact raffaele.calogero@unito.it.
- Video tutorial: https://youtu.be/92RvJe6qqHQ.

## Data Availability

All data are available as part of the github repositories: https://github.com/alessandriLuca/CREDOengine; https://github.com/alessandriLuca/CREDOgui.
